# Multimodal Evaluation and Management of Wagner Syndrome—Three Patients from an Affected Family

**DOI:** 10.3390/genes15091178

**Published:** 2024-09-08

**Authors:** Tomasz Szeligowski, Jasmina Cehajic-Kapetanovic, Shabnam Raji, Ravi Purohit, Hoda Amin, Chetan K. Patel, Kanmin Xue

**Affiliations:** 1 Oxford Eye Hospital, Oxford University Hospitals NHS Foundation Trust, Oxford OX3 9DU, UK; 2 Nuffield Laboratory of Ophthalmology, Nuffield Department of Clinical Neurosciences, University of Oxford, Oxford OX3 9DU, UK; 3 Hull University Teaching Hospitals NHS Trust, Hull HU1 3SA, UK; 4 Great Ormond Street Hospital for Children, London WC1N 1EH, UK

**Keywords:** Wagner syndrome, Versican, inherited retinal disease, retinal detachment, vitreoretinopathy

## Abstract

Wagner syndrome is a rare autosomal dominant vitreoretinopathy caused by mutations in chondroitin sulphate proteoglycan 2 (CSPG2)/Versican (VCAN). Here, we present a retrospective case series of a family pedigree with genetically confirmed Wagner syndrome (heterozygous VCAN exon 8 deletion), as follows: a 34-year-old mother (P1), 12-year-old daughter (P2), and a 2-year-old son (P3). The phenotype included early-onset cataract (P1), optically empty vitreous with avascular membranes (P1, 2), nasal dragging of optic nerve heads associated with foveal hypoplasia (P1, 2), tractional retinoschisis on optical coherence tomography (P2), and peripheral circumferential vitreo-retinal interface abnormality resembling white-without-pressure (P3) progressing to pigmented chorio-retinal atrophy (P1, 2). P2 developed a macula-off retinal detachment, which was treated initially with encircling band + vitrectomy + gas, followed by vitrectomy + heavy silicone oil tamponade for re-detachment from new inferior breaks. Strong vitreo-retinal adhesion was noted intraoperatively, which prevented the separation of posterior hyaloid beyond the equator. Electroretinograms from P1&2 demonstrated attenuated b-waves, a-waves, and flicker responses in light- and dark-adapted conditions, suggestive of generalised retinal dysfunction. Our patients demonstrated the clinical spectrum of Wagner syndrome, highlighting nasal dragging with foveal disruption as a distinguishing feature from other inherited vitreoretinopathies. Surgical outcomes demonstrate significant challenges in managing vitreo-retinal traction and need for further research into strategies to prevent sight loss.

## 1. Introduction

Wagner syndrome is a rare autosomal dominant vitreoretinopathy with a predicted prevalence of less than 1:1,000,000 [1]. It is caused by mutations in the chondroitin sulphate proteoglycan 2 (CSPG2)/Versican (*VCAN*) gene on chromosome 5, which encodes Versican—a widely expressed glycoprotein with diverse roles in human tissues thanks to its modular structure with multiple splice variants and its versatile binding properties [2,3]. During development, it has been shown to play a key role in regulating local concentrations of growth factors, thus directing cell migration and differentiation [4]. In adult tissues, aside from its structural role as a component of the extracellular matrix, it has also been shown to regulate physiological processes such as inflammatory reactions, wound healing, and blood vessel formation [5]. In the eye, Versican is predominantly found in the vitreous where it plays a key structural role through its ability to interconnect components of the extracellular matrix using its terminal domains [6]. However, the full extent of its expression and functions in the eye remains unclear. The main clinical features of Wagner syndrome include optically empty vitreous, avascular vitreous bands, cataracts, and pigmented chorio-retinal atrophy, although other features have also been described with variable consistency [2]. The presence of abnormal vitreo-retinal adhesions in Wagner syndrome results in retinal traction which can lead to retinal detachment—the key surgical complication of this condition. Vitrectomy with retinopexy and/or scleral buckle remain the mainstay treatment modality for these patients; however, ongoing abnormal vitreo-retinal adhesions present a significant surgical challenge and risk factor for further detachment. Due to the rarity of Wagner syndrome, its full clinical spectrum and optimal management strategies are still poorly understood. Here, we present a multimodal clinical assessment of three patients from an affected family to enhance our understanding of this condition.

## 2. Materials and Methods

This is a retrospective case series of a family pedigree affected by Wagner syndrome. All data were obtained through routine clinical care. For the adult patient (P1), informed written consent for publication was obtained directly from the patient. For the two siblings, parental informed written consent was obtained on the children’s behalf with both parents present during the consenting process. For each patient, Optomap ultra-widefield scanning laser ophthalmoscopy images (Optos Plc, Dunfermline, UK) were obtained from both eyes. Where possible, this was supplemented with spectral-domain optical coherence tomography (OCT) and near-infrared reflectance (NIR) imaging (Spectralis HRA-OCT, Heidelberg Engineering, Heidelberg, Germany) of the macula, optic discs, and other areas of interest determined on examination. Full-field electroretinography (ERG) was performed using the RETeval (LKC Technologies, Gaithersburg, MA, USA) handheld ERG device with skin electrodes, following the International Society for Clinical Electrophysiology of Vision (ISCEV) standard protocols [7]. The genetic diagnosis was determined by whole genome sequencing (Illumina, San Diego, CA, USA) and analysis of variants prioritised by the Genomics England bioinformatics pipeline for the NHS Genomic Medicine Service ‘Retinal Disorders (R32.2)’ gene panel. Proband-parent analysis indicates that the *VCAN* exon 8 deletion is maternally inherited. Imaging and ERG data were analysed independently by the authors. Genomic data were obtained from the GeneCards database [8].

## 3. Results

**Figure 1:**
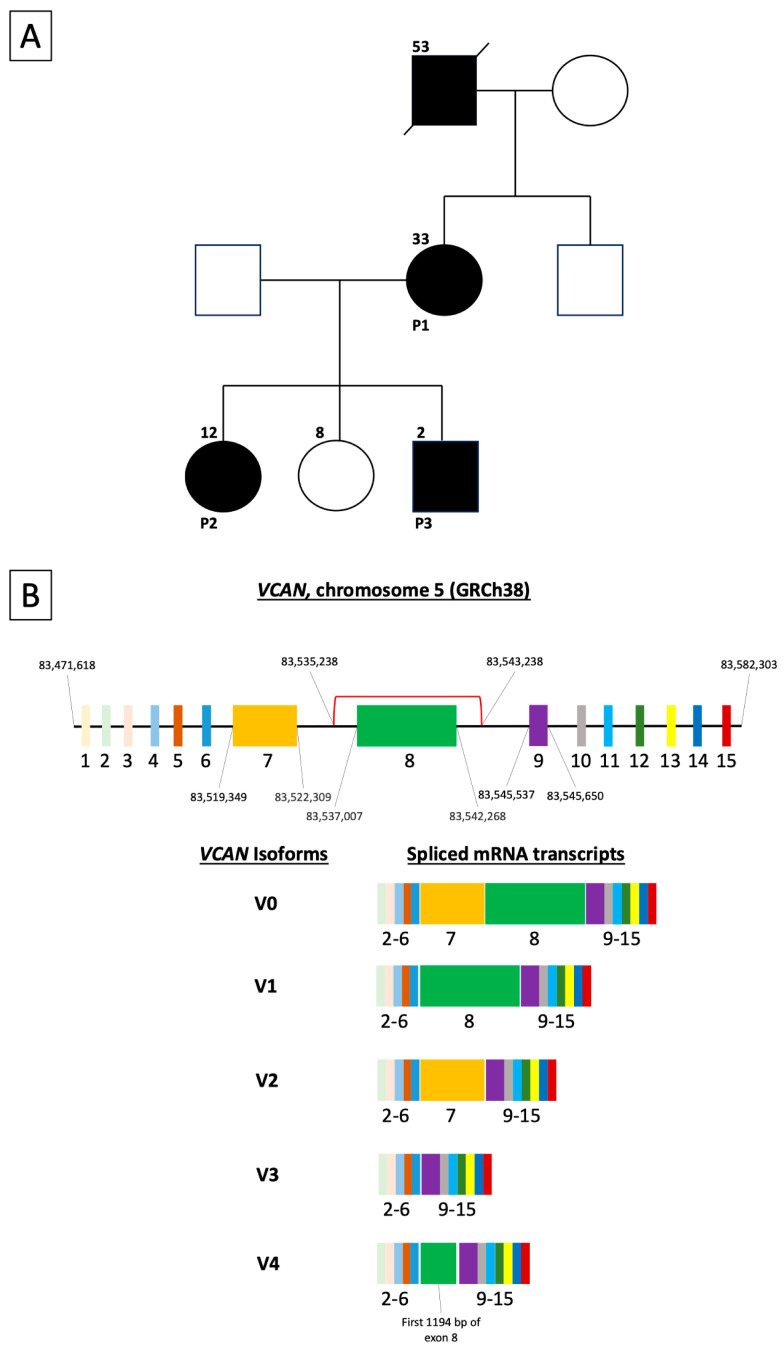
Pedigree of the family affected by Wagner syndrome, and genomic coordinates of the mutation identified in these patients. (**A**) Patient 1 (P1, 33-year-old female) has two children affected by Wagner syndrome—a daughter aged 12 (P2) and a son aged 2 (P3). She also has an unaffected daughter aged 8. The father of P1 was given a diagnosis of ‘choroideremia’ and was blind bilaterally from an early age. The pedigree indicates the autosomal dominant inheritance of Wagner syndrome in this family. (**B**) Schematic representation of the *VCAN* gene exon structure and Versican isoforms. Coloured boxes represent exons of the *VCAN* gene. The region affected by the deletion Chr5(GRCh38):g.83535238-83543238del is shown by the red square bracket, which encompasses the entire exon 8 with consequent loss of isoforms V0, V1, and V4.

The family pedigree can be seen in Figure 1A. Patient 1 is a 33-year-old Caucasian female affected by Wagner syndrome. She has two affected children—a daughter aged 12 (Patient 2) and a son aged 2 (Patient 3)—and one unaffected daughter. The father of all three children is of Afro-Caribbean ethnicity and is unaffected. Genetic testing for all affected patients revealed a heterozygous deletion Chr5(GRCh38):g.83535238-83543238del resulting in deletion of exon 8 in the *VCAN* gene. The genomic coordinates of this deletion and its impact on Versican isoforms [9] are shown schematically in Figure 1B. Her mother, brother, and partner are unaffected, while her deceased father was noted to have been diagnosed with ‘presumed choroideremia’ and was blind bilaterally from an early age. The pedigree presented in Figure 1 indicates an autosomal dominant inheritance pattern of Wagner syndrome in this family, consistent with the previous literature [2]. 

**Figure 2:**
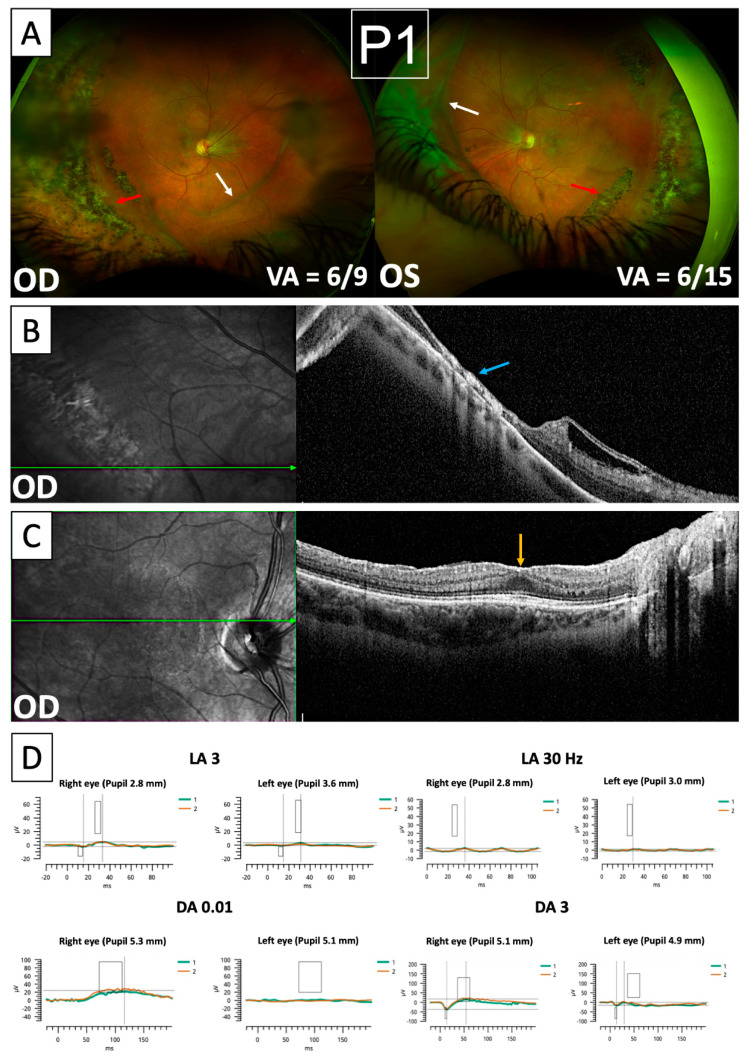
Clinical features of Wagner syndrome exemplified by Patient 1. (**A**) Optomap images showing bilateral nasalisation of retinal bloods vessels, vitreous bands (white arrows), and extensive peripheral pigmented chorio-retinal atrophy (red arrows). (**B**) NIR imaging and OCT through areas of pigmented atrophy show profound thinning and disruption of retinal architecture (blue arrow). (**C**) NIR imaging and OCT through the fovea showing foveal hypoplasia (orange arrow). (**D**) ERG recordings demonstrate bilateral attenuation of a-wave, b-wave, and flicker responses under both light-adapted (**top row**) and dark-adapted (**bottom row**) conditions. The rectangles overlain on each waveform indicate the range of normative values for amplitude and implicit time.

Ultra-widefield imaging of Patient 1 revealed the bilateral presence of avascular vitreous bands characteristic of Wagner syndrome (Figure 2A), which were associated with retinal traction on fundal examination. Furthermore, significant nasalisation of retinal blood vessels was seen in both eyes along with extensive areas of peripheral pigmented chorio-retinal atrophy (Figure 2A). OCTs through areas of pigmentation (Figure 2B) reveal profound thinning and disruption of retinal architecture affecting all layers, indicating severe retinal atrophy. An OCT B-scan through the fovea (Figure 2C) shows foveal hypoplasia with a diminished foveal pit and persistence of inner retinal layers. Profound retinal dysfunction was also evident in ERG recordings obtained from Patient 1 (Figure 2D). The light-adapted responses showed significantly reduced amplitudes and delayed implicit times in both eyes. The dark-adapted responses showed borderline reduced a-waves and b-waves with delayed implicit times in both eyes, although the dark-adapted 0.01 ERG responses were not measurable in the left eye. There was also asymmetry in the recordings, with the left eye showing a greater deficit in responses. Clinically, the patient has bilateral high myopia, reduced best-corrected visual acuity (BCVA), and had previously undergone bilateral cataract surgery followed by YAG laser capsulotomy. She has not, however, developed any vitreo-retinal surgical complications of Wagner syndrome. She also has no significant past medical history and no systemic features. 

**Figure 3:**
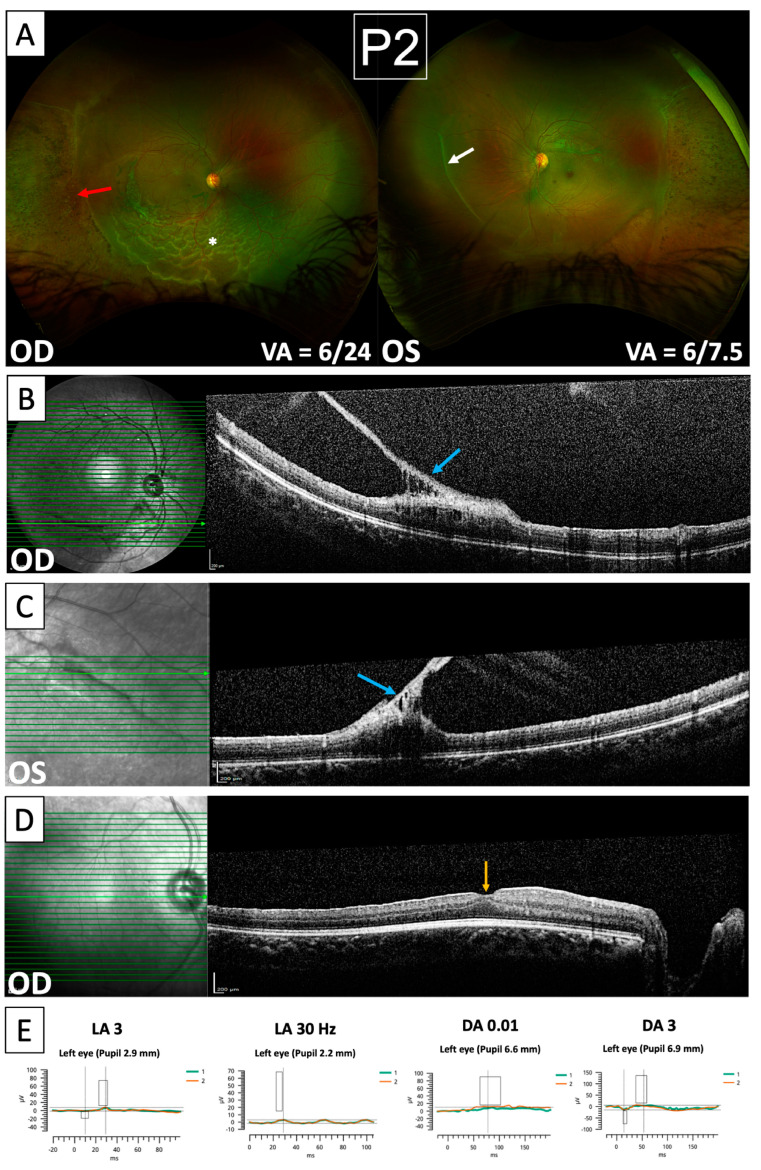
Retinal detachment as a complication of Wagner syndrome in Patient 2. (**A**) Optomap images showing inferior retinal detachment in the right eye (white asterisk), vitreous bands (white arrow), peripheral pigmented chorio-retinal atrophy (red arrow), and nasalisation of retinal blood vessels. (**B**,**C**) NIR imaging and OCT scans of the right eye (**B**) and left eye (**C**) taken five years prior to presentation with retinal detachment showing a vitreous veil exerting retinal traction at its insertion point with developing tractional retinoschisis (blue arrows). (**D**) NIR imaging and OCT scan through the fovea showing foveal hypoplasia (orange arrow). (**E**) ERG recordings showing attenuation of a-waves, b-waves, and flicker responses under light-adapted (LA) and dark-adapted (DA) conditions. The rectangles overlain on each waveform indicate the range of normative values for amplitude and implicit time.

Patient 2 presented with a macula-off retinal detachment in the right eye (Figure 3A). She initially underwent combined encircling band and vitrectomy/endolaser/gas tamponade, but presented with re-detachment one month later from new retinal breaks inferiorly. This was treated with vitrectomy/heavy silicone oil (Densiron 68, Fluoron GmbH, Ulm, Germany) tamponade. During surgery, a strong 360-degree vitreo-retinal adhesion immediately posterior to areas of chorio-retinal atrophy was noted, which precluded the separation of the posterior hyaloid beyond the mid-periphery. At her most recent follow-up (5 months post repeat surgery), her retina remained flat under Densiron with no further complications and improving BCVA in the right eye (6/24 at presentation compared to 6/18 at follow-up). Imaging in this patient revealed a similar pattern of abnormalities to her mother, with nasalisation of retinal blood vessels, the presence of avascular vitreous bands, and areas of peripheral pigmented chorio-retinal atrophy (Figure 3A). OCTs taken 5 years prior to the onset of retinal detachment demonstrate bilateral vitreous bands (Figure 3B,C), which exert significant traction at their points of insertion leading to tractional retinoschisis. The finding of vitreous bands exerting retinal traction is a significant risk factor for further detachment in both eyes in this patient, thus requiring careful monitoring. Similarly to her mother, OCT through the fovea (Figure 3D) showed evidence of foveal hypoplasia. ERG recordings from Patient 2 (Figure 3E) also showed significantly reduced amplitudes with delayed implicit times in light- and dark-adapted conditions; however, only recordings from the left eye could be performed in her case. Similarly to her mother, she has no other past medical history and no systemic features.

**Figure 4:**
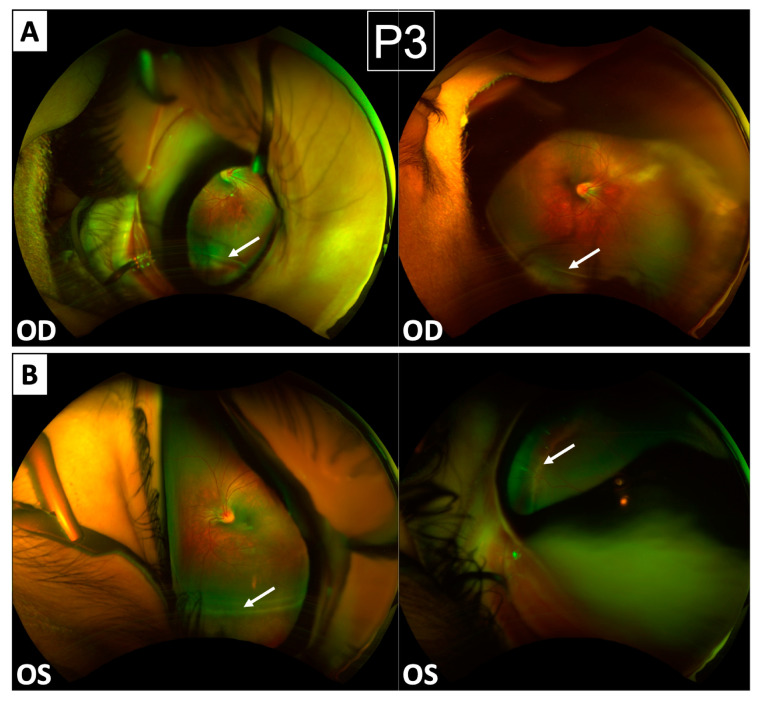
**Early features of Wagner syndrome in Patient 3.** (**A,B**) Optomap images show bilateral nasalisation of retinal vessels along with peripheral non-perfusion. Circumferential white lines (white arrows) were noted bilaterally and may represent developing vitreo-retinal interphase pathology.

Patient 3 was referred originally due to strabismus, which has since been corrected with spectacles. Although views of his retina on imaging were limited due to his age, they demonstrate nasalisation of retinal blood vessels similar to Patients 1 and 2 (Figure 4). Furthermore, circumferential areas resembling white-without-pressure were noted, which likely represent early vitreo-retinal interface pathology, which will evolve towards the pattern of vitreous bands and pigmented atrophy seen in Patients 1 and 2. Although this boy’s visual acuity could not be tested formally, he is able to fix and follow binocularly. At present, he is being managed conservatively. He has no significant medical history and is otherwise developing appropriately for his age, with no systemic features. 

## 4. Discussion

The three patients presented in this study demonstrate the clinical spectrum of Wagner syndrome, from the early manifestations seen in the three-year-old child to later complications seen at age 12 and 33 in the same family. Their clinical findings are consistent with previous reports, but also include features which have previously been under-reported and thus deserve particular mention. First, we noted a pathognomonic pattern of retinal vessel nasalisation in all three patients, which could be related to tractional forces exerted by abnormal vitreo-retinal adhesions, and which is an early feature, as indicated by its presence in Patient 3. To our knowledge, this has only been described in one previous study, which was a follow-up of the original Wagner syndrome cohort [10]. Second, we also observed varying degrees of foveal hypoplasia in Patients 1 and 2, with diminished foveal pit and OCT appearance of pre-retinal membrane. This finding has also been noted in one previous report, and may contribute to the reduced visual acuity seen in Wagner syndrome [11]. Finally, Patient 3 exhibited peripheral retinal changes resembling white-without-pressure, which may represent an early manifestation of vitreo-retinal interface abnormalities. These are likely to develop into 360-degree confluent areas of pigmented chorio-retinal atrophy that are seen in Patients 1 and 2. Similar changes have been previously seen in a 9-year-old patient with Wagner syndrome [12]. Given that the features described above have rarely been reported in the literature to date, more research is needed to establish their prevalence, functional impact, and prognostic value in Wagner syndrome.

ERG recordings obtained from our patients show profound attenuation of a-wave, b-wave, and flicker responses under both light- and dark-adapted conditions, indicating broad retinal cell dysfunction. Notably, the cone-driven light-adapted responses were attenuated to a greater degree, which may indicate a more significant impact on cone function. This level of impairment is probably more than one would expect from the role of pathogenic *VCAN* variants in vitreo-retinal adhesions, suggesting a more direct role in photoreceptor dysfunction and degeneration. Previous studies also showed generalised ERG abnormalities in Wagner patients, including multifocal ERG recordings showing reduced macular cone function [1,2,10,13]. However, there have been inconsistent reports of predominant deficits of light- and dark-adapted recordings, while two studies suggested that the pattern of ERG changes is dependent on the patient’s age [1,10]. 

The retinal abnormalities present in our and others’ patients with Wagner syndrome point towards an essential role of *VCAN* in retinal development and function. This is further indicated by studies showing *VCAN* expression in all layers of both the developing and adult retina, with particularly strong staining in the outer plexiform layer and photoreceptor inner/outer segments [14,15,16]. Furthermore, murine *Vcan* knockout models show profound disruption of outer retinal layers and retinal detachments even in heterozygous models, consistent with the autosomal dominant inheritance pattern of Wagner syndrome [17]. To date, nearly all mutations identified in Wagner syndrome patients were found to cause deletion or skipping of exon 8, and this is also the case in our patients [18]. Exon 8 is the largest exon of *VCAN* and encodes one of its major glycosaminoglycan-binding domains (β-GAG domain) [19]. It is essential for expressing three of its five isoforms (V0, V1, and V4), indicating their importance in the eye (Figure 1B). Previous studies suggested that the abnormalities seen in Wagner syndrome may stem either from insufficiency of the affected isoforms in the eye, or over-expression of the remaining isoforms with deleterious effects [18,20,21], which is in keeping with previous observations that the isoforms of *VCAN* have distinct and tissue-dependent roles [22,23]. Given exon 8 encodes the β-GAG domain which is responsible for binding chondroitin sulphate side chains, its deletion may directly impair the interactions of Versican with other components of the extracellular matrix of the vitreous gel, thus explaining the vitreo-retinal interphase abnormalities seen in Wagner syndrome [24]. However, it may be insufficient to explain other features found in this condition. It is thus clear that in order to understand the pathophysiology of Wagner syndrome, more research is needed to investigate the specific roles that *VCAN* and its isoforms play in the development and function of the human eye. 

Due to the rarity of Wagner syndrome, there is little research available on optimal management strategies for retinal detachment as a complication of this condition. The presence of strong vitreo-retinal adhesions makes surgery challenging and is linked to a significant risk of recurrent tractional-rhegmatogenous retinal detachment. In the case of Patient 2, revision surgery with heavy silicone oil tamponade was required to achieve stabilisation of the retina; however, she remains at risk of re-detachment in both the operated and the fellow eye. One possibility that warrants consideration may be the use of a prophylactic encircling band to relieve 360-degree peripheral vitreo-retinal traction, thus preventing retinal detachment. To date, there has only been one case report of its successful application in Wagner syndrome [12]. Thus, further studies are needed to establish whether this would be a beneficial approach in the long-term and how to risk-stratify patients who might be suitable for such treatment.

## Data Availability

The original contributions presented in the study are included in the article. further inquiries can be directed to the corresponding author/s.
